# Hepatoprotective activity of Lactéol® forte and quercetin dihydrate against thioacetamide‐induced hepatic cirrhosis in male albino rats

**DOI:** 10.1111/jcmm.18196

**Published:** 2024-03-27

**Authors:** Hebatallah M. Saad, Samah S. Oda, Athanasios Alexiou, Marios Papadakis, Mohamed H. Mahmoud, Gaber El‐Saber Batiha, Eman Khalifa

**Affiliations:** ^1^ Department of Pathology, Faculty of Veterinary Medicine Matrouh University Matrouh Egypt; ^2^ Department of Pathology, Faculty of Veterinary Medicine Alexandria University Abees Alexandria Province Egypt; ^3^ University Centre for Research & Development Chandigarh University Mohali Punjab India; ^4^ Department of Research & Development Funogen Athens Greece; ^5^ Department of Research & Development AFNP Med Wien Austria; ^6^ Department of Science and Engineering Novel Global Community Educational Foundation Hebersham New South Wales Germany; ^7^ Department of Surgery II University Hospital Witten‐Herdecke, Heusnerstrasse 40, University of Witten‐Herdecke Wuppertal Germany; ^8^ Department of Biochemistry, College of Science King Saud University Riyadh Kingdom of Saudi Arabia; ^9^ Department of Pharmacology and Therapeutics, Faculty of Veterinary Medicine Damanhour University Damanhour AlBeheira Egypt; ^10^ Department of Microbiology, Faculty of Veterinary Medicine Matrouh University Matrouh Egypt

**Keywords:** histopathology, Lactéol® forte, liver, quercetin dihydrate, rats, Thioacetamide

## Abstract

Liver cirrhosis is a silent disease in humans and is experimentally induced by many drugs and toxins as thioacetamide (TAA) in particular, which is the typical model for experimental induction of hepatic fibrosis. Thus, the objective of the present study was to elucidate the possible protective effects of lactéol® forte (LF) and quercetin dihydrate (QD) against TAA‐induced hepatic damage in male albino rats. Induction of hepatotoxicity was performed by TAA injection (200 mg/kg I/P, twice/ week) in rats. LF (1 × 10^9^ CFU/rat 5 times/week) and QD (50 mg/kg 5 times/week) treated groups were administered concurrently with TAA injection (200 mg/kg I/P, twice/ week). The experimental treatments were conducted for 12 weeks. Hepatotoxicity was evaluated biochemically by measuring alanine aminotransferase (ALT), aspartate aminotransferase (AST) and gamma‐glutamyl transferase (GGT) in the serum and histopathologically with the scoring of histopathological changes besides histochemical assessment of collagen by Masson's trichrome and immunohistochemical analysis for α‐smooth muscle actin (α‐SMA), Ki67 and caspase‐3 expression in liver sections. Our results indicated that LF and QD attenuated some biochemical changes and histochemical markers in TAA‐mediated hepatotoxicity in rats by amelioration of biochemical markers and collagen, α‐SMA, Ki67 and caspase3 Immunoexpression. Additionally, LF and QD supplementation downregulated the proliferative, necrotic, fibroblastic changes, eosinophilic intranuclear inclusions, hyaline globules and Mallory‐like bodies that were detected histopathologically in the TAA group. In conclusion, LF showed better hepatic protection than QD against TAA‐induced hepatotoxicity in rats by inhibiting inflammatory reactions with the improvement of some serum hepatic transaminases, histopathological picture and immunohistochemical markers.

## 1 INTRODUCTION

The liver is an important organ of the body that has an essential role in the metabolic process. Over the past few decades, morbidity and mortality rates of various types of liver disease have increased all over the world. Liver cirrhosis is a silent disease in which 40% of patients haven't had any symptoms over extended times until the development of the decompensated phase, and when the complications appear, a gradual deterioration happens with the result of death, unless the patient is exposed to final therapy, which is hepatic transplantation.^1^ Thioacetamide (TAA) was first used as a fungicide in orange groves and banned in 1960 due to its hepatotoxic effect. Since then, it has been used for the induction of hepatic failure and carcinogenesis in experimental animals.^2^ TAA injection encouraged the development of periportal fibrosis quickly with larger cirrhotic nodules that are similar to human cirrhosis.^3^

Probiotics are described as live microbes that are improving the health once they are given in sufficient quantities. In most cases, the strain of *Lactobacillus* species has been illustrated to improve liver diseases as probiotics.^4^ Lactéol® forte (LF) is a commercial probiotic and pharmabiotic that consists of *Lactobacillus boucard* (*L. fermentum* and *L. delbrueckii*). Interestingly, new research that worked on the human gut microbiota proved the novel therapeutic effect of microbiota.^5^, ^6^

Currently, a great concern of scientific studies has been directed towards the exploration of phytochemical properties, involving several flavonoids, in the food of curative herbs against acute and chronic diseases.^7^ Quercetin (Qu) (3‐, 5‐, 7‐, 3‐, 4‐pentahydroxyflavone) is one natural flavonoid that accounts for about 75% of human total flavonol consumption and is ubiquitously present in different plants. Qu is reported to have anti‐oxidant, anti‐inflammatory, anti‐fibrotic and anti‐carcinogenic properties.^8^ Additionally, Qu has gotten a lot of concern because of its hepatoprotective properties on alcoholic or non‐alcoholic steatohepatitis by monitoring the genes that are related to fat metabolism and the release of an inflammatory substance, which appeared as decreasing the hepatic lipids accumulation, modulating inflammatory stimuli and preventing liver collagen accumulation and the development of liver cirrhosis.^9^, ^10^, ^11^

Despite the many studies conducted on hepatic cirrhosis and disorders, it is important to discover the benefits of natural products that may decrease the signs and death rate in patients with liver failure. From this point of view, this experimental study was conducted to investigate the possible protective effect of LF and Qu against the hepatic injury induced by TAA injection in rats.

## 2 MATERIALS AND METHODS

### 2.1 Chemicals and drugs

Thioacetamide (TAA) was obtained from LOBA Chemie, Mumbai, India, as creamy white crystals freely soluble in water. It was freshly prepared as a solution in distilled water and stirred well until crystals were dissolved. Lactéol fort (*Lactobacillus LB*) sachets, 10 billion (10^10^ CFU) and Spent culture medium, 160 mg, were obtained from Rameda‐pharmaceuticals Company, Egypt. Each sachet contains *L. LB* corresponding to *L. delbrueckii* and *L. fermentum*. QD was manufactured by SDFCL, India, in the form of yellow powder, which was dissolved in corn oil^12^ and stirred well until the complete dissolution of powder.

### 2.2 Animals, experimental design and sample collection

Thirty healthy adult male albino rats with an initial weight of 100 ± 10 g were purchased from a closed random‐bred colony at the Faculty of Agriculture, Alexandria University and kept in the Faculty of Veterinary Medicine, Matrouh University, Egypt. All animals were acclimatised 2 weeks before the beginning of the study for adaptation and to ensure normal growth and behaviour. Rats were housed in separate clean, disinfected and well‐ventilated polycarbonate cages (5 rats /cage) at a constant room temperature of 25 ± 2°C, relative humidity of 50% ± 5% and at a light/dark cycle of 12 h throughout the experimental period. The animals were given ad libitum access to a standard rodent diet and water. All the mentioned care and experimental design strictly follow the standard roles described by the institutional animal care and use committee (IACUC), Alexanderia University, and the approval number is QRA/0304108/17/2017.

Rats were randomly allocated into six groups (five rats each): Control group: I/P injected with 2 mL/kg BW normal saline twice/week and orally administrated 2 mL distilled water five times/week. TAA group: I/P injected with TAA at a dose of 200 mg/kg BW twice/week as described by Al‐Gayyar et al.^13^ LF group: Orally administrated with lactéol® forte at 1 × 10^9^ CFU/rat five times/week as described by Ghazy et al.^14^ QD group: Orally administrated QD at a dose of 50 mg/kg dissolved in corn oil five times/week as described by Gelen et al.^12^ TAA+ LF treated group: I/P injected with TAA (200 mg/kg BW) simultaneously with lactéal fort that was given orally (1 × 10^9^ CFU/rat). TAA + QD treated group: I/P injected with TAA 200 at a dose of mg/kg BW twice/week concurrently with oral administration of QD at a dose of 50 mg/kg dissolved in corn oil five times/week. Clinical signs and body weights were recorded throughout the experiment. Rats were maintained in their respective groups for 12 weeks. At the end of the 12th week post‐treatment, individual blood samples were collected from the retro‐orbital venous plexus. Blood samples were placed in plain centrifuge tubes, left in slope position at room temperature to clot and centrifuged at 3000 rpm for 15 min for serum separation. The clear serum was carefully separated by automatic micropipette then transferred into clean dry Eppendorf and preserved at −20°C until transferred to lab for biochemical analysis. After euthanasia, livers were carefully excised and washed with the ice‐cold saline buffer to remove the blood then blotted with filter papers then weighed. Livers' weights were recorded, and relative liver weight (RLW) was calculated as follows: RLW = liver weight (g)/final BW (g) × 100.

### 2.3 Biochemical assays

Serum alanine aminotransferase (ALT), aspartate aminotransferase (AST) and gamma‐glutamyl transferase (GGT) were measured according to the manufacturer's instructions using commercial kits provided by bio‐diagnostic Company (Vitro Scient Co., Egypt).

### 2.4 Histopathological and immunohistochemical evaluations

Three liver tissue specimens from each rat were preserved in 10% neutral buffered formalin for at least 24 h and then prepared by paraffine embedding technique^15^ for studying histopathological changes. A semiquantitative lesion score was estimated to show the prevalence and the degree of severity of different hepatic histopathological changes between groups. Five fields of each liver section from each rat/group were randomly examined at ×100 using a light microscope. Then the following scores were used: (−) absence of lesion, (+) mild = 5%–25%, (++) moderate = 26%–50% and (+++) severe = > 50% of examined liver section. For examination of connective tissue, liver sections were processed down to distilled water, mordant in Bouin's solution and stained with Masson's trichrome stain.^16^

For immunohistochemical staining, deparaffinized sections were obtained on positive slides and then hydrated in a graded series of alcohol solutions. Sections were incubated in antigen retrieval (boiling the sections at 98°C for 20 min in 10 mmol/L sodium citrate buffer, pH (6.0) then treated with 3% H_2_O_2_ to block endogenous peroxidase). Monoclonal antibodies for Ki67, α‐smooth muscle actin (α‐SMA) and caspase3 (DAKO Corp.) were applied to the slides and incubated in the humid chamber overnight at 4°C. Secondary biotinylated antibody was then applied, followed by incubation with streptavidin peroxidase. Sections were washed with phosphate buffer saline three times after each step. Sections were stained with diaminobenzidine chromogen solution and counterstained with Mayer's haematoxylin.^17^

### 2.5 Histomorphometry assessment

For histomorphometric quantitative analysis, ten original random micrographs have been captured at a magnification of ×100 per group to calculate the mean area % of the collagen in Masson's trichrome‐stained sections. Also, ten fields at a magnification of ×400 were captured per group to calculate % of diaminobenzidine (DAB)‐stained areas for α‐SMA, ki67 and caspases 3. The digital images were analysed by Image analysis software (Fiji Image J, 1.51n, NIH, USA) according to the method described by Crowe and Yue.^18^

### 2.6 Statistical analysis

Data of biochemical results and image analysis results were analysed by one‐way analysis (ANOVA) with the aid of statistical analysis system software (Version 9.30, User's Guide, SAS Institute Inc., Cary, NC, USA). The significance between different groups was assessed by Duncan. Levels were considered significant at *p* < 0.05. The results are expressed as mean values with their corresponding standard errors. Additionally, we used Shapiro–Wilk test to check the normality and homogeneity of variance.

## 3 RESULTS

### 3.1 Clinical signs and mortalities

No clinical signs were observed in the control and LF groups. While in the QD group, rats showed anorexia and lethargy. The rats in the TAA‐treated group exhibited depression and loss of appetite, and the group TAA + LF showed mild anorexia. Rats of the TAA + QD‐treated group revealed clinical signs identical to those described in the TAA‐treated group. No mortalities were recorded in all groups except TAA‐treated group, which showed one mortality.

### 3.2 Effect on body weight and relative liver weight ratio (RLW)

As noticed in Table 1, the treatment of male albino rats with TAA led to a significant decrease in the BW and a significant elevation in the RLW as opposed to control rats. While LF and QD‐treated rats showed non‐significant alteration in the BW and RLW as compared to control rats. There was a significant reduction in BW and a non‐significant change in RLW in TAA + LF and TAA + QD treated groups compared to control and TAA‐treated rats.

**TABLE 1:** Effect of thioacetamide (TAA), lactéol® forte (LF) and quercetin dehydrate (QD) alone or in combination for 12 weeks on body weight (BW), relative liver weight (RLW) and serum liver enzymes (ALT, AST and GGT) in male albino rats.

Groups	BW (g)	RLW (%)	ALT (U/L)	AST (U/L)	GGT (U/L)
CTR	259.67 ± 7.97^a^	3.10 ± 0.31^b^	57.80 ± 1.62^ab^	137.00 ± 16.17^a^	1.86 ± 0.20^b^
TAA	182.25 ± 17.03^b^	3.78 ± 0.13^a^	86.00 ± 5.20^a^	180.20 ± 19.79^a^	3.91 ± 0.48^a^
LF	249.33 ± 11.05^a^	3.08 ± 0.26^b^	54.30 ± 5.35^b^	140.83 ± 23.14^a^	1.65 ± 0.19^b^
QD	259.00 ± 8.37^a^	3.09 ± 0.12 ^b^	55.58 ± 3.83^b^	136.52 ± 16.77^a^	1.95 ± 0.13^b^
TAA + LF	188.00 ± 7.11^b^	3.20 ± 0.17^ab^	51.31 ± 16.86^b^	161.00 ± 24.37^a^	3.25 ± 1.60^ab^
TAA + QD	185.20 ± 14.43^b^	3.48 ± 0.19^ab^	62.23 ± 0.62^ab^	167.25 ± 21.39^a^	3.08 ± 0.27^ab^

*Note*: Mean ± standard errors. Means bearing different letters within the same column are significant at (*p* < 0.05).

Abbreviations: ALT, alanine aminotransferase; AST, aspartate aminotransferase; CTR, control group; GGT, gamma glutamyl transferase.

### 3.3 Effect on the serum liver enzymes activity (ALT, AST and GGT)

As shown in Table 1, there was no significant changes in serum levels of ALT and AST in all treated groups compared to the control group. Regarding serum levels of GGT, there was a significant elevation in the TAA‐treated group compared to the control group. The remaining groups did not show any significant changes in serum levels of GGT compared to the control group.

### 3.4 Necropsy results

**FIGURE 1:**
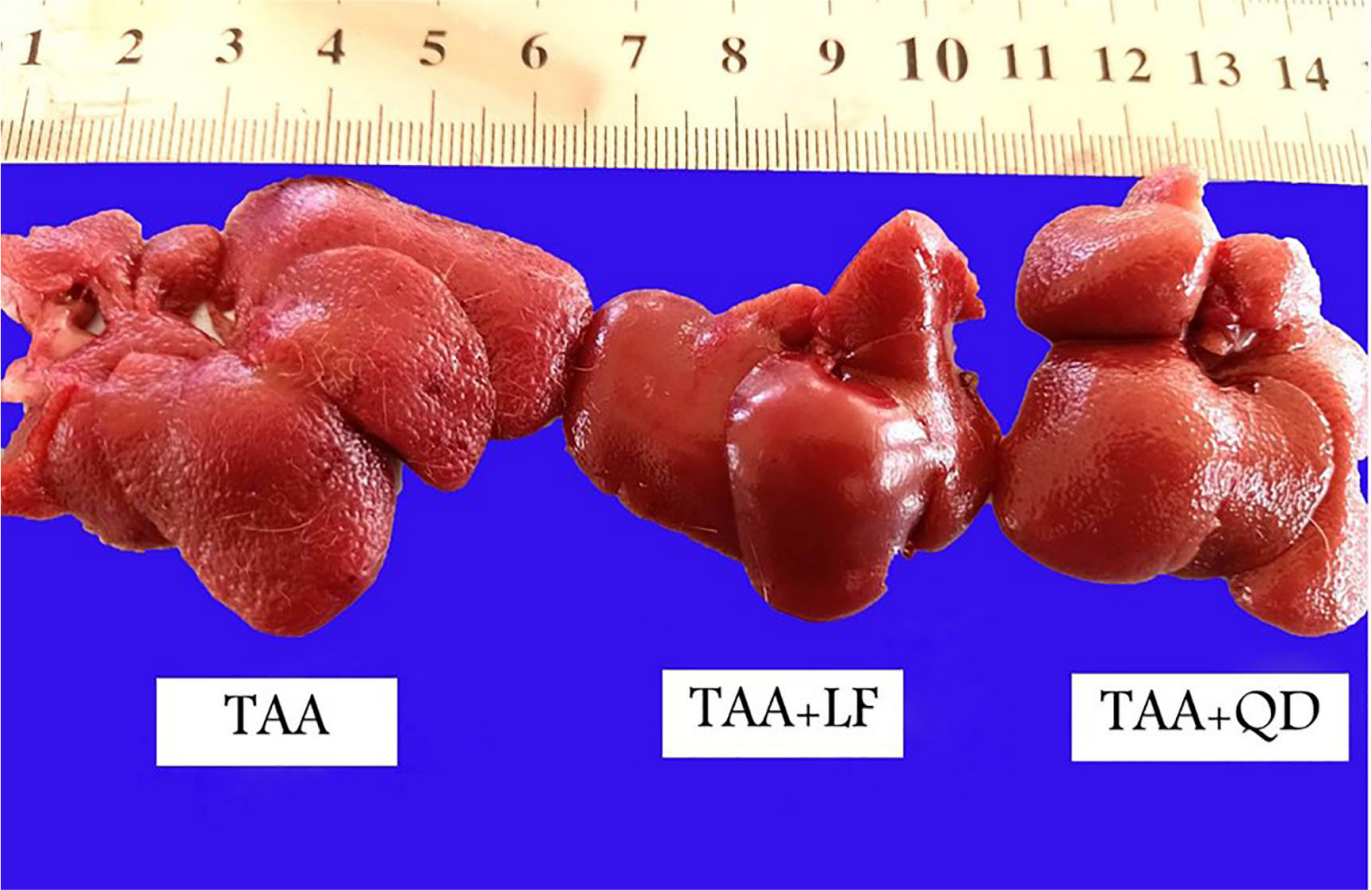
Livers from thioacetamide (TAA) group: Irregular nodular surface; TAA + Lactéol® forte group: Slightly congested liver, smooth surface and focal necrosis; TAA + Quercetin dihydrate group: Pale liver with irregular surface.

Rats' livers of the TAA group had diffused multi‐nodular surface (cirrhotic nodules) and a hard consistency, which decreased in the TAA + LF group more than TAA + QD group (Figure 1). Livers of the LF group resembled the control, but with slight congestion. Livers of most rats in the QD group were enlarged and diffusely pale with a finely reticulated surface in some areas and congestion in another area.

### 3.5 Histopathological examination

**FIGURE 2:**
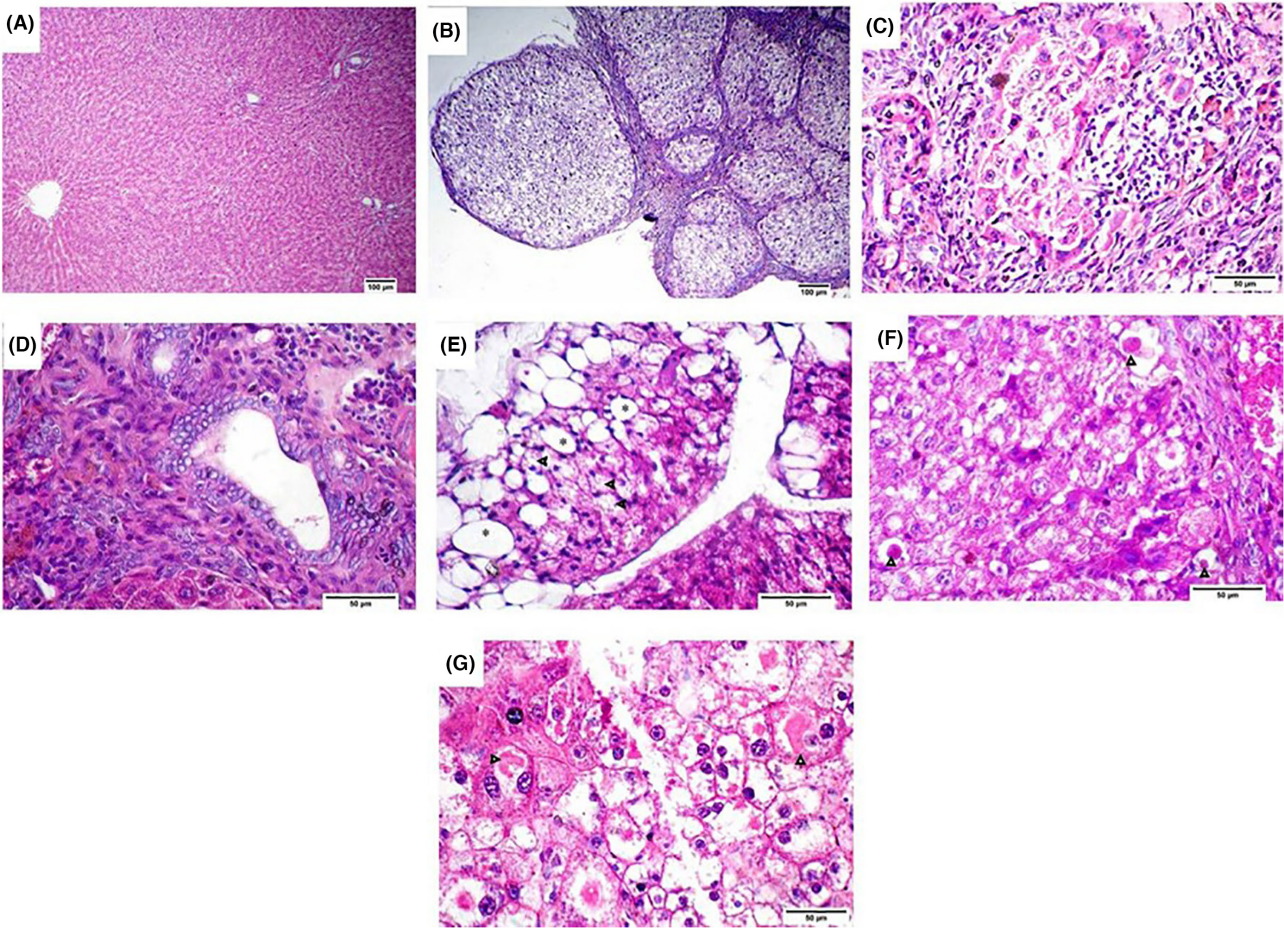
Micrographs of HE stained liver sections from control group (A) Normal hepatic architecture with radially arranged hepatic cords around central veins and normal portal traids, Thioacetamide group (B–G), (B) Disorganization of hepatic parenchyma with extension of fibrous tissue proliferation with formation of parenchymal pseudolobules, (C) Hepatocytes showing variable degrees of necrosis and apoptosis with presence of golden brown hemosiderin pigment and mononuclear cells infiltration, (D) Hyperplastic regenerative oval cells, some collagen bundles and lymphocytic infiltrations besides hyperplasia of biliary epithelium and formation of newly formed bile ductules, (E) Macrovesicular (*) and microvesicular steatosis (arrowheads), (F) Intracellular eosinophilic hyaline globules (arrowheads), (G) Eosinophilic intracytoplasmic inclusions (Mallory‐ like bodies) (arrowheads). Scale bar = 100 μm for (A, B). Scale bar = 50 μm for (C–G).

**FIGURE 3:**
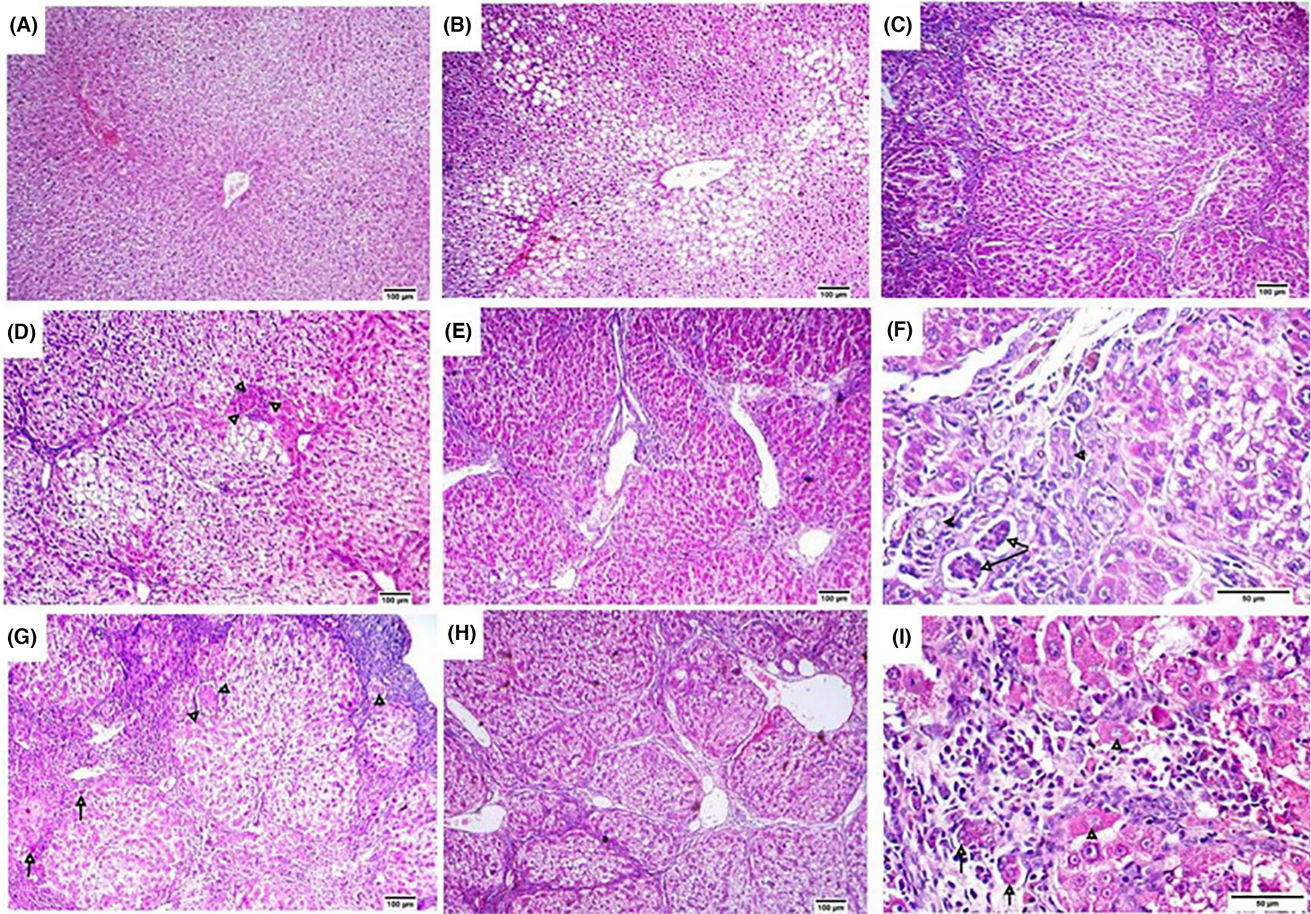
Micrographs of HE stained liver sections from Lactéol® forte group (A, B), (A) Mild hepatocellular vacuolation and mild congestion, (B) Moderate vacuolar degeneration, macrovesicular and microvesicular steatosis, Thioacetamide (TAA) + Lactéol® forte group (C–F), (C) Pseudolobulation with disappearance of central vein, (D) Hepatocellular necrosis (arrowheads) and cytoplasmic vacuolation of hydropic type, (E) Periportal necrosis and fibrosis with mononuclear inflammatory cells, (F) Necrotic cholangitis (arrows), hyperplastic oval cells with formation of nonfunctional bile ductules (arrowheads), mild hemosiderosis and vacuolation, TAA + Quercetin dihydrate group (G–I), (G) Multiple apoptotic bodies (arrows) and multifocal hepatic necrosis (arrowheads), (H) Hepatocytic vacuolation, moderate steatosis and moderate ductular reaction, (I) Regenerating hepatic plates (arrows) besides chronic inflammatory cells, proliferation of stellate cells and anisokaryosis with chromatin dispersion in some hepatocytes (arrowheads). Scale bar = 100 μm for (A–H). Scale bar = 50 μm for (F, I).

Microscopic pictures of haematoxylin and eosin (HE)‐stained liver sections of the control group showed normal hepatic architecture with radially arranged hepatic cords around central veins and normal portal triads (Figure 2A). In the TAA group, there was severe disorganization of hepatic parenchyma with the extension of fibrous tissue proliferation resulting in the formation of parenchymal pseudolobules (Figure 2B). Inside these pseudolobules, hepatocytes showed severe vacuolar degeneration, severe degrees of necrosis and apoptosis associated with the absence of the central vein and with the presence of golden‐brown hemosiderin pigment and mononuclear cell infiltration (Figure 2C). Also, cirrhotic nodules were surrounded by hyperplastic regenerative oval cells, some collagen bundles and lymphocytic infiltrations besides hyperplasia of biliary epithelium and formation of newly formed bile ductules (Figure 2D). Macrovesicular and microvesicular steatosis were also observed (Figure 2E). In addition, intracellular eosinophilic hyaline globules (Figure 2F) as well as eosinophilic intracytoplasmic inclusions (Mallory‐like bodies) were viewed (Figure 2G). Livers of rats in LF group were similar to those of the control group except for mild hepatocellular vacuolation and mild congestion (Figure 3A). In QD‐treated group, there was moderate vacuolar degeneration, macrovesicular and microvesicular steatosis (Figure 3B) and portal lymphocytic infiltrates. Also, few eosinophilic apoptotic bodies and sporadic hepatocellular necrosis were observed. In TAA + LF‐treated group, the recorded lesions were nearly similar to those observed in the TAA‐treated rats but less in severity (Table 2). Most of the hepatic parenchyma showed pseudolobulation with the disappearance of the central vein (Figure 3C), moderate hepatocellular necrosis, moderate cytoplasmic vacuolation of hydropic type (Figure 3D) and periportal necrosis and fibrosis with mononuclear inflammatory cells (Figure 3E). Necrotic cholangitis, hyperplasia of regenerative oval cells, formation of nonfunctional bile ductules, mild hemosiderosis and vacuolation were noticed (Figure 3F). In the TAA + QD‐treated group, the liver of euthanized rats showed fewer lesions than those of the TAA‐treated rats but more severe lesions than those reported in the TAA + LF‐treated group (Table 2). Wherein, hepatic dissociation and expansion of fibrous tissue into hepatic lobules forming pseudolobules were marked, besides moderate lymphocytic infiltrates and multiple apoptotic bodies inside cirrhotic nodules with multifocal hepatic necrosis (Figure 3G). Also, hepatocytic vacuolation, moderate steatosis and moderate ductular reaction were observed (Figure 3H). Regenerating hepatic plates were noticed besides chronic inflammatory cells, the proliferation of hepatic stellate cells and anisokaryosis with chromatin dispersion in some hepatocytes (Figure 3I).

**TABLE 2:** Histopathological scoring for hepatic lesions recorded in male albino rats treated with thioacetamide (TAA), lactéol® forte (LF) and quercetin dehydrate (QD) alone or in combination.

Groups	Lesions					
	Vacuolar degeneration	Steatosis	Necrosis	Apoptosis	Fibroplasia	Ductular reaction
Control	−	−	−	−	−	−
TAA	+++	+++	+++	+++	+++	+++
LF	+	−	−	−	−	−
QD	+	++	+	+	−	−
TAA + LF	++	+	++	++	++	++
TAA + QD	++	++	++	+++	++	++

*Note*: Lesion score: (−) absence of lesion, (+) mild = 5%–25%, (++) moderate = 26%–50% and (+++) severe = >50% of examined liver section.

**FIGURE 4:**
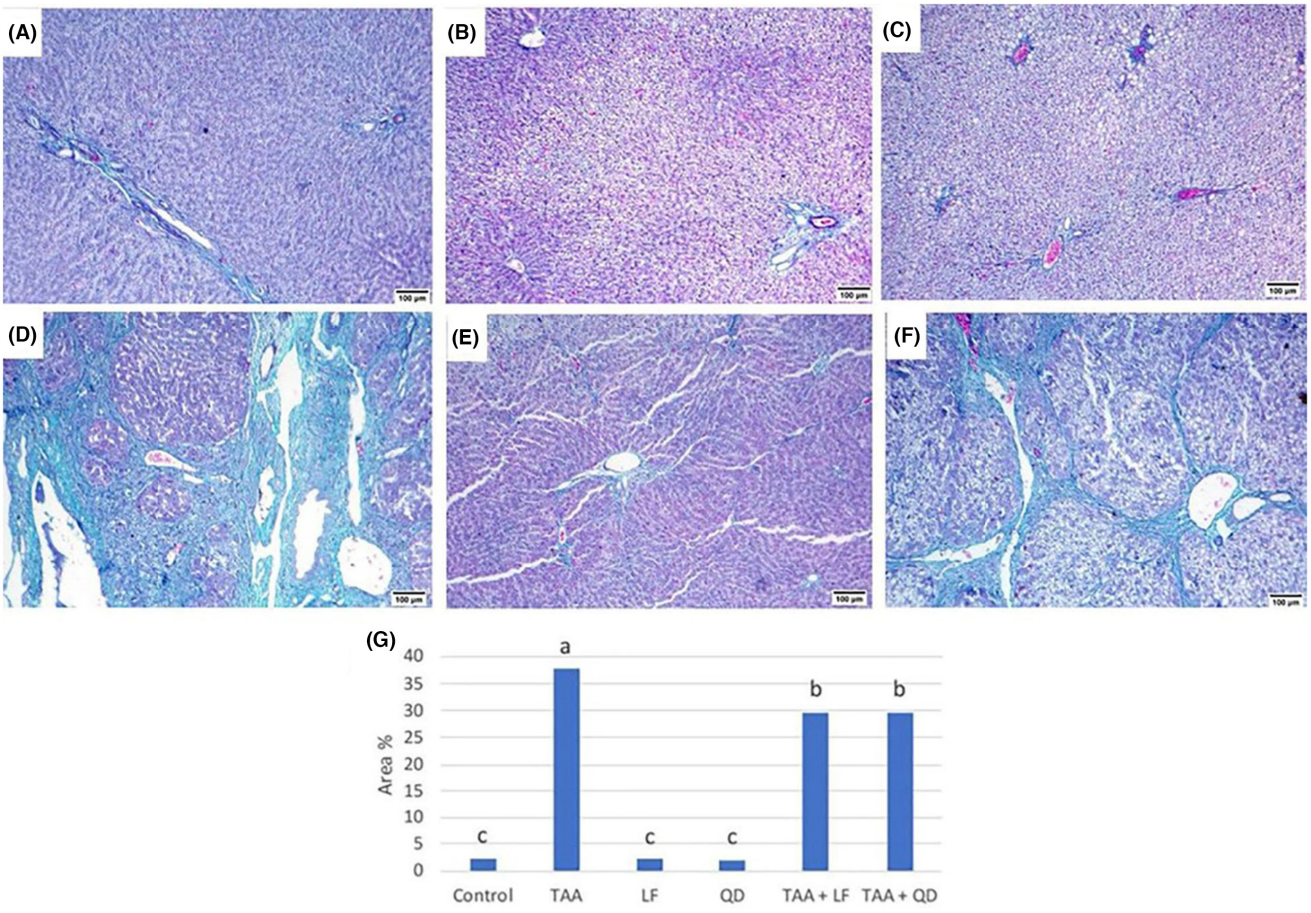
Masson's trichrome stained hepatic sections. (A) Control; (B) Lactéol® forte (LF); (C) Quercetin dehydrate (QD) groups: Normal distribution of green collagen fibres; (D) Thioacetamide (TAA) group: Marked thickening of portal areas with green stained collagen fibres extended to infiltrate the surrounding hepatic parenchyma (bridging fibrosis); (E) TAA + LF; (F) TAA + QD groups: Decreased fibroplasia; (G) Quantitative image analysis of the average area % for Masson's trichrome in different treated groups compared to control one. Columns are group means. Means without a common letter differ significantly (*p* < 0.05). Scale bar = 100 μm.

Masson's trichrome‐stained liver sections of control (Figure 4A), LF (Figure 4B) and QD (Figure 4C)‐treated rats showed normal distribution of green collagen fibres around central veins, portal area and sinusoids. While the TAA‐treated group exhibited marked thickening of portal areas with green‐stained collagen fibres extended to infiltrate the surrounding hepatic parenchyma, forming pseudolobules and bridges to central and portal veins (Figure 4D). Also, pericellular hepatic fibrosis was noticed. Compared to the TAA‐treated group, collagen deposition was decreased in the TAA + LF (Figure 4E) than the TAA + QD (Figure 4F) treated group. Quantitative analysis displays that LF and QD groups showed non‐significant alterations, and the group revealed a significant elevation in area % of Masson's trichrome staining when matched to the control group (Table 3, Figure 4G). Regarding to TAA + LF and TAA + QD groups, area % of Masson's trichrome staining was significantly reduced compared to the TAA group (Table 3, Figure 4G).

**TABLE 3:** Effect of thioacetamide (TAA), lactéol® forte (LF) and quercetin dehydrate (QD) alone or in combination for 12 weeks on the average area % of histochemical stains expression in male albino rats.

Groups	Masson's trichrome	α‐SMA	Ki67	Caspase3
CTR	2.28 ± 0.25^c^	0.96 ± 0.12^b^	0.10 ± 0.04^c^	0.07 ± 0.02^c^
TAA	37.75 ± 2.60^a^	9.20 ± 1.25^a^	25.08 ± 4.57^a^	31.15 ± 2.99^a^
LF	2.14 ± 0.34^c^	0.90 ± 0.17^b^	0.20 ± 0.03^c^	0.06 ± 0.02^c^
QD	2.08 ± 0.24^c^	0.61 ± 0.26^b^	3.32 ± 0.92^c^	6.62 ± 2.18^c^
TAA + LF	29.75 ± 2.80^b^	7.71 ± 0.52^a^	14.78 ± 2.71^b^	23.08 ± 2.80^b^
TAA + QD	29.69 ± 1.92^b^	8.92 ± 1.09^a^	20.81 ± 2.55^ab^	34.44 ± 2.84^a^

*Note*: Mean ± standard errors. Means bearing different letters within the same column are significant at (*p* < 0.05).

Abbreviations: α‐SMA, α‐smooth muscle actin; CTR, control group.

### 3.6 Immunohistochemistry analysis

**FIGURE 5:**
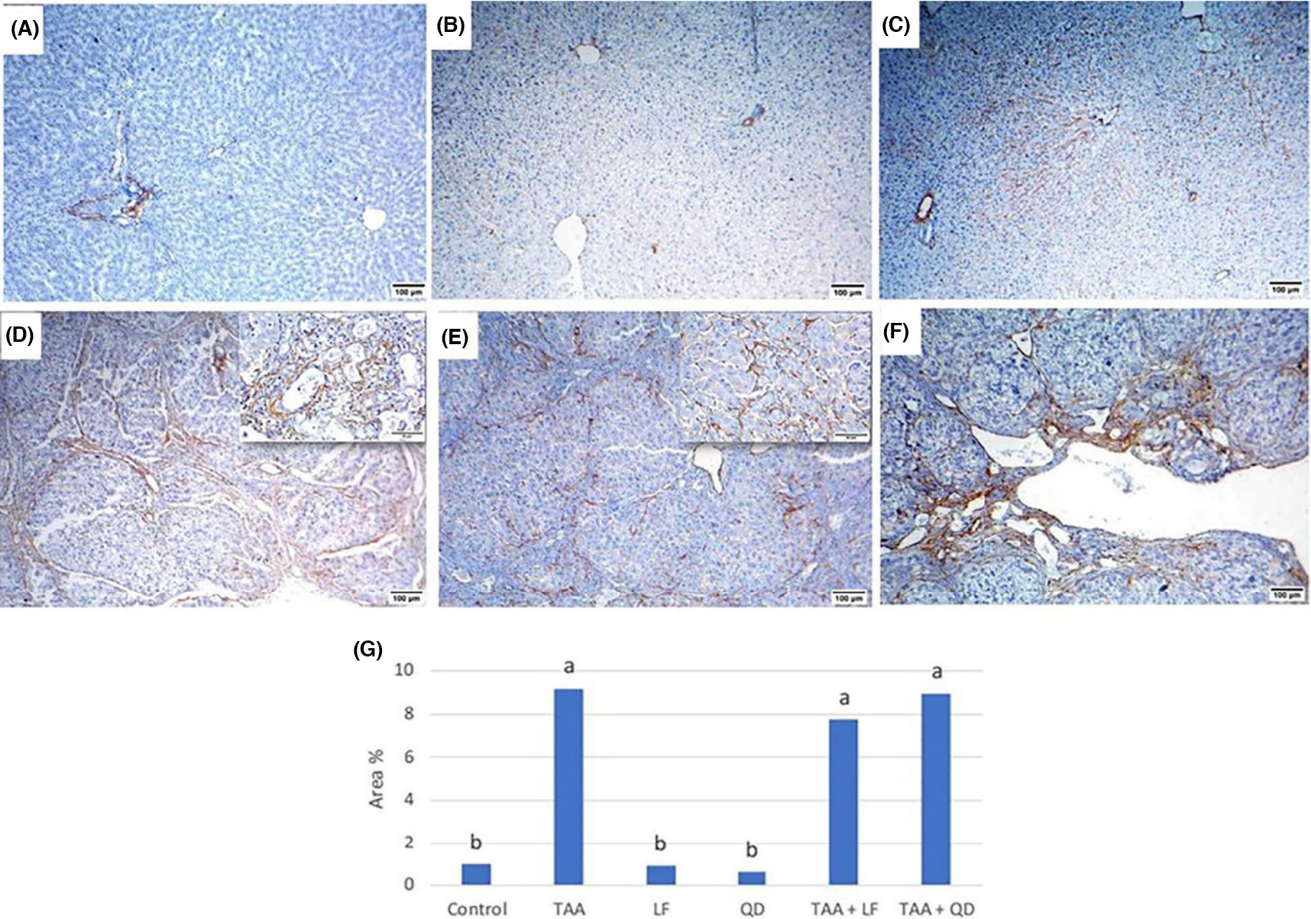
α‐SMA immunohistochemical analysis of rats' livers. (A) Control; (B) Lactéol® forte (LF); (C) Quercetin dehydrate (QD) groups: Normal minimal brown staining; (D) Thioacetamide (TAA) group: Marked brown immunostaining for myofibroblasts, inset showing portal and periductal positive brown staining of activated hepatic stellate cells (HSCs); (E) TAA + LF group: Decreased expression of activated HSCs, inset: Perisinusoidal positive brown staining (F) TAA + QD group: Decreased expression of activated HSCs; (G) Quantitative image analysis of the average area % for α‐SMA immunoexpression in different treated groups compared to control one. Columns are group means. Means without a common letter differ significantly (*p* < 0.05). Scale bar = 100 μm for (A–F), Scale bar = 50 μm for (inset D, E).

Livers of control (Figure 5A), LF (Figure 5B) and QD (Figure 5C) rats showed slight normal expression of α‐SMA expressed as brown staining around the portal and central veins where myofibroblasts were present. While the liver of TAA‐treated rats showed intense brown immunoexpression of α‐SMA‐stained myofibroblasts, which were highly detected alongside the fibrous septa, portal areas and perisinusoidal spaces (Figure 5D). Furthermore, α‐SMA‐positive immunostaining was less expressed in TAA + LF (Figure 5E) than TAA + QD (Figure 5F)‐treated rats. Statistically, the area % of α‐SMA positive immunoexpression in LF and QD groups showed a non‐significant elevation compared to control rats, while the TAA group revealed a significant elevation in area % of α‐SMA positive immunoexpression (Table 3, Figure 5G). In addition, no significant difference was noticed in the TAA + LF and TAA + QD groups compared to the TAA group (Table 3, Figure 5G).

**FIGURE 6:**
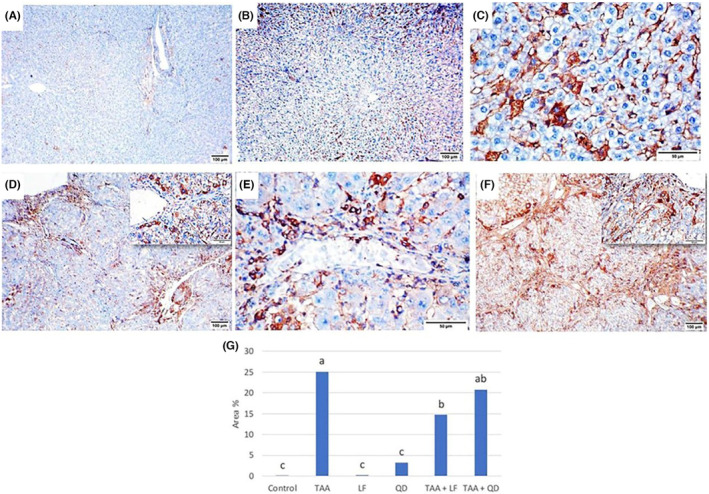
Ki67 immunohistochemical analysis of rats' livers. (A) Control; (B) Lactéol® forte (LF); (C) Quercetin dehydrate (QD) groups: Minimal to moderate brown immunostaining; (D) Thioacetamide (TAA) group: Marked brown immunostaining, inset: Brown immunostaining of hepatocytes cytoplasm; (E) TAA + LF; (F) TAA + QD groups: Decreased Ki 67 immunoexpression, inset: Perisinusoidal positive brown staining; (G) Quantitative image analysis of the average area % for Ki67 immunoexpression in different treated groups compared to control one. Columns are group means. Means without a common letter differ significantly (*p* < 0.05). Scale bar = 100 μm for (A, B, D, F) and scale bar = 50 μm for (C, E, inset D, F).

Livers of control (Figure 6A) and LF (Figure 6B) rats showed minimal Ki67 immunoexpression in the cytoplasm and nuclear membrane of a few hepatocytes. Regarding to QD‐treated group, ki67 was moderately expressed as brown immunostaining in the cytoplasm of hepatocytes in QD‐treated group (Figure 6C). In contrast, strong Ki67 expression in the TAA group mostly in hepatocytes cytoplasm and fewer in nuclear membrane (Figure 6D) was observed. Additionally, Ki67 was less expressed in TAA + LF (Figure 6E) than in TAA + QD (Figure 6F)‐treated rats. Quantitative analysis of the area% of Ki67 immunoexpression revealed a non‐significant elevation in LF and QD groups and a significant rise in the TAA‐group compared to the control group (Table 3, Figure 6G). Compared to the TAA group, the TAA + LF group exhibited a significant reduction in the area% of Ki67 immunoexpression, while QD group showed a non‐significant decrease (Table 3, Figure 6G).

**FIGURE 7:**
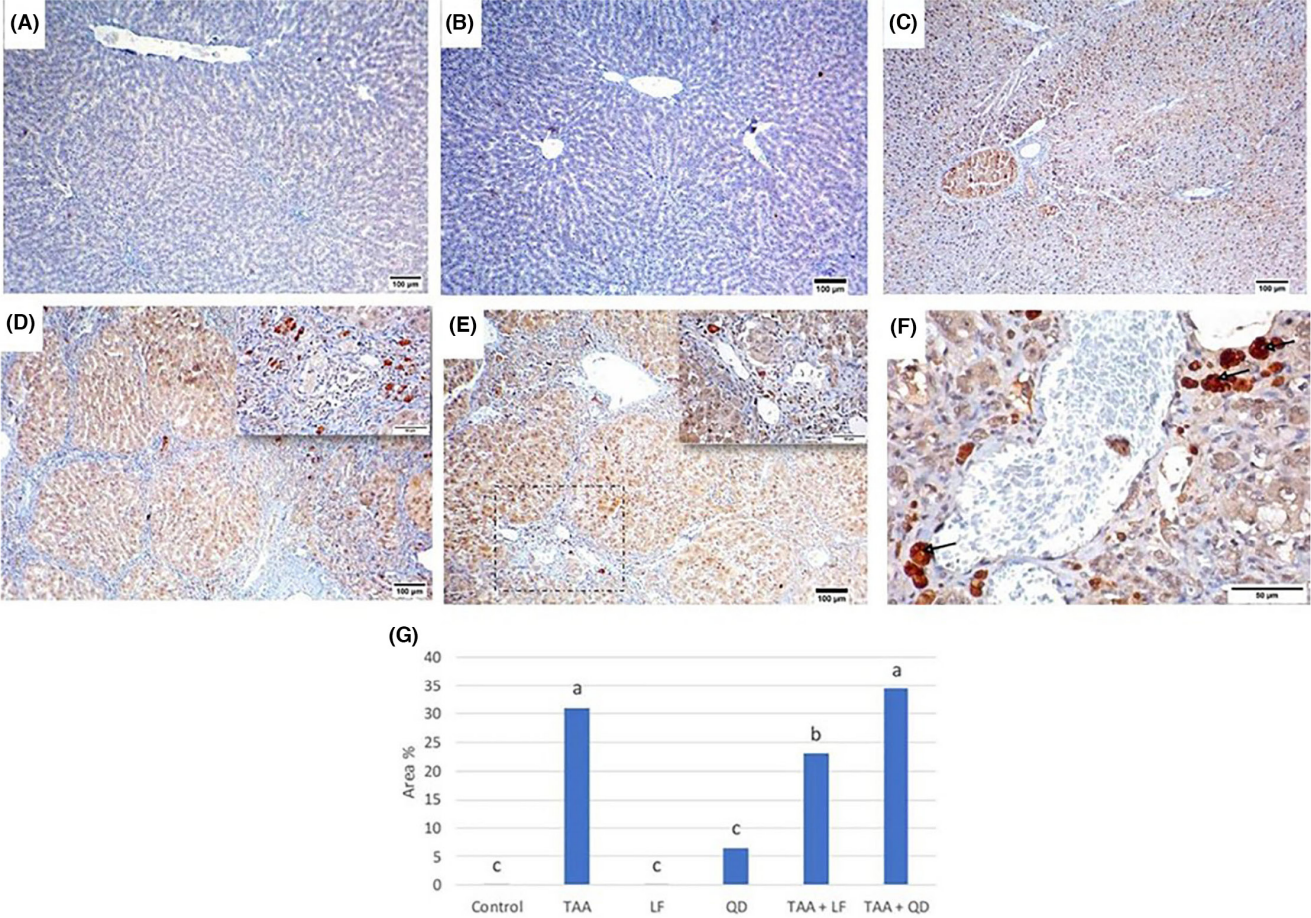
Caspase 3 immunohistochemical analysis of rats' livers. (A) Control; (B) Lactéol® forte (LF) groups: Negative antibody reaction; (C) Quercetin dehydrate (QD) group: Minimal brown immunostaining; (D) Thioacetamide (TAA) group: Diffuse brown hepatocellular staining, inset: Portal multifocal brown cytoplasmic staining; (E) TAA + LF group: Decreased expression of Caspase 3, inset: Minimal portal hepatocellular cytoplasmic staining; (F) TAA + QD group: Multifocal hepatocellular cytoplasmic staining (arrows); (G) Quantitative image analysis of the average area % for caspase 3 immunoexpression in different treated groups compared to control one. Columns are group means. Means without a common letter differ significantly (*p* < 0.05). Scale bar = 100 μm for (A–E), Scale bar = 50 μm for (F, inset D, E).

Regarding immunohistochemical analysis for caspase 3 in the control (Figure 7A) and LF (Figure 7B) rats group, no brown positive reaction was detected either in the cytoplasm or nucleus (Figure 7A) while QD‐treated group, caspase 3 was faintly expressed in the cytoplasm of hepatocytes (Figure 7C). In the TAA group, a strong positive reaction in the cytoplasm of hepatocytes was recorded (Figure 7D). The LF‐treated group showed immunoexpression similar to that detected in the control group. In addition, caspase 3 was less expressed in TAA + LF treated rats (Figure 7E) while QD treatment did not reverse the caspase 3 expression (Figure 7F) when compared with the TAA group. Compared to the TAA group, the TAA + LF group exhibited a significant reduction in the area% of caspase 3 immunoexpression, while the TAA + QD group showed a non‐significant change (Table 3 and Figure 7G).

## 4 DISCUSSION

The induction of hepatic damage through TAA injection is one of the experimental methods widely used to identify hepatoprotective agents due to its outstanding solubility in the water, and a prolonged injury and recovery pattern, giving significant time to study mechanisms.^19^ TAA‐induced liver cirrhosis is considered to be a typical model because it mimics many aspects of the human disease that lead to the development of fibrosis, cirrhosis and liver cancer in rats.^20^, ^21^ Qu is a member of the flavonoid family and more specifically a subclass called flavonol and is widely distributed in the plant kingdom. In the mouth, Qu released from the food can interact with salivary protein and form soluble Qu‐protein binary aggregates.^22^ In the stomach, Qu is exposed to the strong acidic condition and may be degraded to phenolic acids by bacterial ring fission, leading to the breakdown of Qu.^23^ In the small intestine, there is efficient glucuronidation of Qu by the action of uridine diphosphate glucuronosyltransferases and extensive O‐methylation of Qu by the action of catechol‐O‐methyltransferase.^24^ After absorption, it reaches the peaks at approximately 30 min before its metabolized by glucuronidation and sulfation.^25^ Subsequently, those Qu and Qu derivatives are transported by the hepatic portal vein to the liver, where they are further metabolised, including O‐methylation, sulfation and glucuronidation.^26^ The conjugation of Qu with sulfate is carried out by sulfotransferases. When Qu is O‐methylated, its major products are 30‐O‐methylquercetin (isorhamnetin) and 40‐O‐methylquercetin (tamaraxetin) to a lesser extent. The resulted Qu is released into blood circulation via the portal vein of the liver.^25^ The kidney is a major organ of excretion, and perhaps benzoic acid derivatives are a common metabolite of Qu.^27^ Thus, our study aimed to illustrate the possible protective efficiency of LF and Qu against the hepatocellular injury induced by TAA injection in rat model.

Our results showed a significant decrease in body weight with a significant increase in the RLW ratio week in TAA‐treated group. This may be attributed to TAA causing marked toxicity in rats, which interferes with the body weight gain. Previous studies attributed the same outcome to lower levels of nutrient absorption and metabolic efficiency after exposure to TAA.^28^, ^29^ Furthermore, TAA + LF and TAA + QD showed non‐significant differences in body weight as compared to TAA‐treated rats. Also, one mortality has been recorded in the TAA group, and this finding is parallel to previous research.^3^ In our results, despite the induced hepatic cirrhosis in animals of group II, the level of serum ALT and AST showed a non‐significant increase from the control value. Also, the same result was obtained by Palacios et al.^30^ after 16 weeks of TAA injection in C3H/He mice. On the contrary, Salama et al.,^31^ Đurašević et al.^32^ and Ayoub et al.^33^ found a significant increase in serum ALT after injection of TAA. Additionally, Ogaly et al.^34^ and Abdel‐Rahman et al.^35^ showed a substantial increment in the serum levels of ALT and AST after intraperitoneal injection of TAA at a dose of 100 mg/kg 3 times for 2 weeks. Additionally, Abdel‐Rahman et al.^36^ detected a substantial increment in activities of AST and ALT with a reduction in albumin and total protein levels after TAA injection for 14 days (5 or 10 mg/kg, orally). This variation may be due to the strain of animals, the dose of TAA and/or conditions of the experiment. While serum levels of GGT were significantly increased as compared to the control group. These results were in agreement with Salama et al.,^31^ El‐Gendy et al.^37^ and Ayoub et al.^33^ The combined administration of TAA with LF and QD showed a non‐significant decrease in serum hepatic enzymes indicating that both may have a protective effect against TAA hepatotoxicity. The downregulating effect of LF and QD against serum‐level enzymes was reported by Bahr et al.^38^ and Afifi et al.,^39^ respectively. Our necropsy results of the TAA‐treated group revealed diffuse, multinodular (cirrhotic nodules) and hard inconsistency. The same results were obtained by Gao et al.,^40^ De Souza et al.^41^ and Shaban et al.^2^ Additionally, Ayoub et al.^33^ showed that the administration of TAA at a dose of 100 mg/kg for 8 weeks resulted in marked hepatocellular necrosis, intense inflammatory cell proliferation and portal fibroplasia with pseudo‐lobulation. Furthermore, the liver of TAA + LF or TAA + QD‐treated rats was less cirrhotic. Microscopic observations indicated severe hepatocellular vacuolation and centrilobular necrosis and fibrosis that progressed into bridging fibrosis then cirrhotic nodules of different sizes with intense mononuclear inflammatory cells, marked oval cells hyperplasia and ductular reaction. Cytoplasmic Mallory bodies were seen inside the cytoplasm of most hepatocytes as also described by Roomi et al.^42^ According to previous studies, CYP2E1 is a major contributor to TAA bioactivation and toxicity, which leads to the generation of sulfine and sulfone metabolites.^43^ TASO (sulfine) is responsible for karyomegally, changing cell permeability and increasing intracellular Ca levels that initiate apoptosis. Furthermore, TASO2 binds to hepatic macromolecules as proteins and lipid‐producing centrilobular necrosis so that TAA, unlike most hepatotoxins that produce periportal hepatocellular necrosis. The histopathological changes were in harmony with Al‐Attar and Al‐Rethea,^24^ Shareef et al.^21^ and Abdel‐Rahman et al.^35^, ^36^ Lesions in TAA + QD‐treated group were lesser in the extent than those of the TAA‐treated rats but most severe than those reported in the TAA + LF‐treated group. The findings of QD treatment came in accordance with Afifi et al.^39^ and Salama et al.^31^ In the present work, the administration of LF resulted in the improvement of the histoarchitecture of the liver with increasing the reactivity of inflammatory and immune cells against TAA‐harmful effects as documented previously by Bahr et al.^38^ Moreover, Moro‐García et al.^44^ and Garcia‐Castillo et al.^45^ showed the immunostimulant effect of *L. delbrueckii* and *L. fermentum*, respectively.

The process of extracellular matrix (ECM) production is initiated by the activation and transformation of quiescent hepatic mesenchymal cells (as hepatic stellate cells (HSCs) and portal fibroblasts) into myofibroblasts that express α‐SMA.^46^ HSCs are found in space of Disse under certain cytokine‐mediated pathways that undergo principle morphologic changes involving the loss of lipid vacuoles and are transformed into a spindle‐shaped myofibroblast that replicates at the site of injury to produce collagen and other ECM component as proteoglycans, fibronectin and hyaluronan and thus propagate fibrosis.^47^ Moreover, fibrosis in the portal tracts is primarily generated by portal fibroblasts rather than HSCs.^48^ In our study, TAA‐treated group showed marked upregulation of fibrotic markers (collagen by Masson's trichrome and α‐SMA expression) in comparison to the control rats. The same result was obtained by Đurašević et al.^32^ and El‐Gendy et al.^37^ Furthermore, Abdel‐Rahman et al.^35^ showed that the intraperitoneal injection of TAA (100 mg/kg) for 2 weeks resulted in a substantial increment in hepatic fibrosis markers as TGF‐β1, collagen I, connective tissue growth factor (CTGF), focal adhesion kinase (FAK) and α‐SMA. LF showed antifibrotic activity as demonstrated by the down‐regulation of α‐SMA‐stained myofibroblast and collagen expression by Masson's trichrome.^38^ Also, QD decreased the increment of collagen deposition and α‐SMA expression by TAA, but its effect was lower than LF. The anti‐fibrotic effect of QD is reported by Wang et al.^49^ and Li et al.^50^

Ki67 is a nuclear protein antigen and is widely used as a proliferative marker for the growth and proliferation of cancer cells so it is important for cancer grading and prognostic evaluation.^51^ Ki67 is associated with active cell proliferation and expressed in all phases of the cell cycle (G1, S, G2 and M phases), except in resting cells (G0), with a sharp decrease after mitosis.^52^ In the present study, the cytoplasmic and nuclear staining of Ki67 was markedly increased in the TAA‐treated group as compared to the control group. Similar findings were documented by Köhn‐Gaone et al.^53^ and Salama et al.^31^ Ki67 expression in the cytoplasm and cell membrane of an unknown mechanism was reported in breast cancer by Faratian et al.,^54^ in mice after fumonisin toxicosis by Sozmen et al.^55^ and in Wistar rats after malathion toxicity by Baiomy et al.^56^ The present study showed that LF can counteract the TAA‐induced increment in ki67 expression. These results agree with Kahouli et al.^57^ who reported a decrease in Ki67 expression in *L. fermentum* and *L. acidophilus* probiotic mixture against colorectal cancer in mice as well as with Aindelis and Chlichlia^58^ who documented the anti‐proliferative effect of probiotic in which Lactobacillus decreased expression of Ki67. Furthermore, TAA + QD‐treated group showed a non‐significant decrease in the Ki67 immunoexpression as compared to TAA‐treated group. Lu et al.^59^ reported that quercetin reduced Ki67 expression significantly, and the combination with docetaxel, a chemotherapy agent, showed the strongest inhibitory effect on tumour cell proliferation in docetaxel‐resistant prostate cancer.

The apoptosis pathway induced by TAA depends on the activation of caspase 3.^60^ In our study, the high rate of apoptosis detected in the TAA group is supported by previous studies.^31^, ^61^ Moreover, caspase 3 was less expressed in TAA + LF‐treated rats when compared with the TAA group. Our findings come in line with Chen et al.^62^ who reported that Lactobacillus restored the increased expression of caspase‐3‐positive hepatocytes. Conversely, QD treatment did not reverse the caspase‐3 expression when compared with the TAA group. In parallel, Choi and Kim^63^ stated that oral administration of quercetin (100 and 250 mg/kg BW for 18 days) in mice leads to its accumulation in the liver with subsequent apoptotic body formation, which is evidenced by increased caspase‐3 expression. Additionally, Wang et al.^64^ observed that quercetin sensitises doxorubicin‐induced apoptosis in liver cancer cells mainly triggered by increasing the loss of the mitochondrial membrane potential, indicating the increase in the mitochondrial breakdown, that enhanced release of cytochrome c and the increase in caspase 3 subsequently. Quercetin has pro‐apoptotic potential through diverse mechanisms via its antioxidant properties and the inhibition of the p53 gene and BCL‐2 protein.^65^ Additionally, Thangasamy et al.^66^ showed that the administration of quercetin in the diet of a rat model demonstrated a decrease in the occurrence of mammary tumour initiation by carcinogens. The capacity of quercetin to inhibit tumour growth exhibits a dose‐dependent impact, whereby lower concentrations provide chemopreventive effects while higher concentrations possess pro‐oxidant properties or potential direct therapeutic attributes. Furthermore, Spencer et al.^67^ showed that quercetin and its O‐methylated metabolites induced neuronal death by inhibiting Akt/PKB and ERK survival pathway and activating the c‐Jun N‐terminal kinase death pathway.

## 5 CONCLUSION

Overall, our data suggest that TAA intoxication induced deleterious effects on body weight, biochemical parameters and histopathological and immunohistochemical pictures of the liver of male albino rats which can be ameliorated by LF or QD. LF showed better hepatic protection than QD against TAA toxicity in the improvement of some serum hepatic transaminases, histopathological picture and immunohistochemical markers. Hence, LF can be used as a promising candidate to protect against fibrosis. Also, because of the discrepancy in different studies, further research work is required to better understand the potential risk or safety of dietary QD.

## AUTHOR CONTRIBUTIONS

**Hebatallah M. Saad:** Conceptualization (equal); methodology (equal); writing – original draft (equal); writing – review and editing (equal). **Samah S. Oda:** Writing – original draft (equal). **Athanasios Alexiou:** Visualization (equal); writing – original draft (equal). **Marios Papadakis:** Resources (equal); validation (equal); visualization (equal). **Mohamed H. Mahmoud:** Writing – review and editing (equal). **Gaber El‐Saber Batiha:** Writing – original draft (equal). **Eman Khalifa:** Methodology (equal); supervision (equal).

## CONFLICT OF INTEREST STATEMENT

There is no conflict of interest.

## ARRIVE GUIDELINES

The authors confirm that the study was carried out in compliance with the ARRIVE guidelines.

## Data Availability

All data generated and analysed during our study are included in this article.
